# Managing Relationship Decay: Network, Gender, and Contextual Effects

**DOI:** 10.1007/s12110-015-9242-7

**Published:** 2015-10-21

**Authors:** Sam B. G. Roberts, R. I. M. Dunbar

**Affiliations:** Department of Psychology, University of Chester, Chester, UK; Department of Experimental Psychology, University of Oxford, Oxford, UK

**Keywords:** Gender differences, Relationship maintenance, Personal networks, Family, Friendship, Emotional closeness

## Abstract

Relationships are central to human life strategies and have crucial fitness consequences. Yet, at the same time, they incur significant maintenance costs that are rarely considered in either social psychological or evolutionary studies. Although many social psychological studies have explored their dynamics, these studies have typically focused on a small number of emotionally intense ties, whereas social networks in fact consist of a large number of ties that serve a variety of different functions. In this study, we examined how entire active personal networks changed over 18 months across a major life transition. Family relationships and friendships differed strikingly in this respect. The decline in friendship quality was mitigated by increased effort invested in the relationship, but with a striking gender difference: relationship decline was prevented most by increased contact frequency (talking together) for females but by doing more activities together in the case of males.

Social relationships play an especially important role in our social arrangements and, both historically in recent evolutionary time and in contemporary traditional and postindustrial societies, have very significant fitness consequences. The quality of social relationships that individuals maintain with others is related to psychological well-being (Furukawa et al. 1998; Kawachi and Berkman 2001), and both primates (Silk et al. 2009) and humans (Holt-Lunstad et al. 2010; Oesch and Dunbar 2015) show well-established links among social relationships, morbidity, and mortality. Examining individual differences in the strategies people use to maintain different types of ties, and the cost-benefit trade-offs between these different types of ties, may provide valuable insight into the adaptive significance of successfully managing social relationships (Baumeister and Leary 1995; Sutcliffe et al. 2012; Nettle et al. 2013).

Understanding why relationships exist (their functions), the costs incurred in maintaining them (an essential component in the cost-benefit basis of all evolutionary explanations), and how and why they fail (despite the benefits they provide) is as relevant to relationships in small-scale societies as to those in postindustrial societies, since all are likely to be underpinned by the same deep psychological mechanisms irrespective of cultural differences in their surface structure between societies. Longitudinal studies can disentangle cause and effect more effectively than cross-sectional studies and can examine how specific life events that involve physical or social separation affect the stability of relationships (Crosnoe 2000). Such transitions shed light on the costs involved in maintaining different types of relationship across time and space. Transitions put friendships under pressure for two reasons. First, once people become separated by geographical distance, they have to make an active effort to meet with or contact old friends. Second, moving to a new location provides opportunities to make new friends, which takes time and energy that could otherwise be devoted to maintaining old friendships.

One theoretical model that has frequently been used to understand what happens to social relationships during periods of transition is the Relationship Investment Model (Rusbult 1983) (e.g., Oswald and Clark 2003; van Duijn et al. 1999). According to this model, commitment to a relationship is a function of satisfaction with the relationship plus investments into the relationship minus the possible alternatives to the relationship. Moving away may decrease satisfaction with old relationships because of an increase in the availability of alternatives (new, potentially more attractive friends) and an increase in maintenance costs (when physically separated).

However, the Relationship Investment Model does not explicitly recognize the possible constraints on the number of social relationships that can be maintained at a particular level of emotional intensity. The notion of constraints on network size has its origins in two divergent areas of research. It was raised early on in the study of social networks (Bernard and Killworth 1973; Pool and Kochen 1978), but the precise nature of these constraints and how they influence social relationships has not been fully explored in the field of social network analysis (Roberts 2010). In contrast, the notion of constraints on the number of social relationships individuals can maintain is a central feature of the social brain hypothesis (Dunbar 1992, 1998). This model argues that the key selection pressure driving the evolution of large brains in both primates and humans is the complexity of managing long-term social relationships in a stable social group (Dunbar and Shultz 2007). In primates, relationships are maintained by grooming a small set of key allies, but the amount of time primates can devote to grooming is limited by competing demands for time (Dunbar et al. 2009). Because of this, the proportion of the day available for grooming is limited, placing an upper limit on the size of primate groups (Lehmann et al. 2007).

Human relationships also have a tendency to weaken (Burt 2000, 2002; Cummings et al. 2006). To prevent this decay requires time-consuming maintenance behaviors, principally communication and joint activities (Cummings et al. 2006; Oswald and Clark 2003). Since time is an inelastic resource (Nie 2001), this need to maintain relationships places an upper limit on the number of relationships that can be maintained at each level of emotional intensity (Roberts 2010; Sutcliffe et al. 2012). Whilst the implications of this time constraint have been extensively explored in primates (Lehmann et al. 2007), much less is known about how it acts to limit the number and quality of relationships humans can maintain with others (Roberts 2010; Sutcliffe et al. 2012).

Although social psychology has a long-standing interest in studying how social relationships change (Feld et al. 2007; Suitor et al. 1997), the focus of this research has been limited almost entirely to very close relationships (parent-offspring, romantic partner, best friend). Yet we have many more kinds of relationships than just these, including those with extended kin and with friends of different degree, and these types have hardly ever been studied. Social networks are far from being homogenous; rather they consist of a number of different types of ties that vary in emotional quality. Granovetter (1973) suggested that “the strength of a tie is a (probably linear) combination of the amount of time, the emotional intensity, the intimacy (mutual confiding) and the reciprocal services which characterize the tie” (1973:1361). The well-known dichotomy of “strong” and “weak” was only presented as a “rough, intuitive basis” (1973:1361) for categorizing ties.

Nonetheless, although this conceptualization of ties has proved useful in certain respects—for example, the effect of weak ties on areas such as job searches (Granovetter 1983)—this approach typically fails to recognize that the differences between ties of different strengths are not simply linear. Instead, qualitative differences in types of ties result in distinct groupings of ties at similar levels of emotional intensity or contact frequency (Hill and Dunbar 2003; Sutcliffe et al. 2012; Zhou et al. 2005). In effect, social networks consist of a series of relationship circles (each in turn split evenly between family and friends: Dunbar et al. 2014), with each successive circle containing more individuals than the one inside it but having relationships of declining emotional quality (Hill and Dunbar 2003; Roberts and Dunbar 2011; Sutcliffe et al. 2012; Zhou et al. 2005). These circles bear a remarkably consistent scaling relationship to each other, with successive circles being ~3 times larger than the one inside it (Zhou et al. 2005), a relationship also noted in the social structure of other mammals that have multilevel social systems (Hill et al. 2008). The relevance of this for the nature of friendships, and for the pattern of changes through time in relationships, has not as yet been widely appreciated (Sutcliffe et al. 2012).

In addition to the strength of the tie, an important factor in how relationships change is the distinction between family and friends. Hamilton’s rule of kin selection (Hamilton 1964) states that behavior toward others should be influenced by the relative costs and benefits of the behavior, weighted by the coefficient of genetic relatedness. In line with this rule, people are more likely to help kin than friends, and this help is less contingent on the personal relationship between the two individuals (Curry et al. 2013; Espinoza 1999; Madsen et al. 2007; Wellman and Wortley 1990). In terms of the relationship between contact frequency and emotional closeness, affinal kin follow exactly the same relationship rules as genetic kin (Burton-Chellew and Dunbar 2011), and we therefore include genetic and affinal kin in the same category (kin).

In this study, we examined the changes in relationship quality for a group of high school students making the transition from school to university or work. This transition represents one of the major life transitions and thus provides a particularly acute example of the processes of interest to us. We extend previous research in this area in three key ways. First, we recruited the students whilst they were still at school, so we have information on their social relationships before they left school, as well as what happened to these relationships in the year after they left school. In contrast, most previous studies (Berman and Sperling 1991; Hays and Oxley 1986; Kenny 1987; Oswald and Clark 2003; Paul and Brier 2001) only started when the participants were already at university. Retrospective studies of this kind risk overlooking some changes. Second, previous longitudinal work has focused on a relatively small number of strong ties (Oswald and Clark 2003; Wellman et al. 1997), yet weak ties also have a tendency to decay (Burt 2000; Milardo and Wellman 1992). Moreover, decay has typically been studied simply in terms of whether ties are named in the network at successive time periods (Burt 2000; Morgan et al. 1997). In this study, we examined how the emotional quality of ties within the entire active network changed through time. Third, we explicitly explore the effect of distance on how social relationships change during this period. Around half the participants stayed in their hometown to go to university or to work, and half went to university elsewhere. Thus, we are able to disentangle whether leaving the hometown causes the relationships to change or simply the opportunities provided by a different social environment. In this study, we examine the effect of proximity on relationships with both friends and relatives.

We define the active network as all relatives plus all unrelated individuals with whom the participant feels that they have a genuine, personal relationship (Roberts et al. 2009; Sutcliffe et al. 2012). Thus we are able to explore how relationships with both close and distant friends, and *all* family members, change during the transition from school to university or work. Although our sample size is modest, this should be set against the fact that we track all these individuals and all their relationships in considerable detail across 18 months. To the best of our knowledge, this is the first time such an extensive personal network has been studied prospectively during a life transition. Studying the entire active personal network allows for the effects of constraints to be examined more extensively because there may be trade-offs between different parts of the social network (for example, old school friends versus new university friends; close friends versus more distant friends).

We test five hypotheses as to how the ties in the personal network will be affected by the transition from school to university. First, previous studies have shown that family relationships appear to be more resilient than others to the life transitions (e.g., Burt 2000; Kenny 1987; Sullivan and Sullivan 1980; Pipp et al. 1985; van Duijn et al. 1999), and we test whether this is so in our sample: we predict that friendships will decay more in terms of a decrease in emotional closeness than relationships with family members (H1). Second, we examine the effect of distance on the decay in these relationships. Distance makes it difficult to maintain friendships and also makes visits back to their hometown more costly in terms of both travel time and money. We thus predict no effect of staying or leaving the hometown on the strength of relationships with family, but we do predict a greater decay in friendships for leavers than for students that stay in their home town to go to university (H2). Third, we predict that there will be an effect of relationship strength on the tendency of the relationship to decay (H3). Thus, we expect relationships in the “inner” layer of the network (i.e., relatives who are genetically more closely related and friends whom the participant has known for longer) to be more resistant to decay. Fourth, we predict that participants who add more friends to the network will show a greater decline in closeness to existing friends since the time and energy invested into the new friends comes at the expense of time and energy that has been put into maintaining old friendships (H4). Finally, we predict that increased interaction with the person concerned is necessary to maintain a relationship after the transition; otherwise relationship quality will deteriorate (H5).

## Methods

### Participants

Thirty students (15 females; average age = 18.1 ± 0.48 *SD*, range 17–19 years) who were in the final year of high school were recruited for the study. All the participants lived in the same large city in England. On average, participants had lived in the city for 189.0 ± 47.2 *SD* months (~16 years). The city is ethnically diverse, and in keeping with this diversity, 17 of the participants were white, 11 were of Pakistani Asian origin, and 2 were of black African origin.

Participants were asked to complete detailed questionnaires on their complete active personal network at the beginning of the study (T1), and then at two further time points: 9 months (T2) and 18 months (T3: end of first calendar year at university, or equivalent). Of the 30 participants who started the study at T1, 29 (96.7%) completed the questionnaire at T2, and 25 (83.3%) did so at T3. A total of 25 participants (12 females; 15 white, 8 Pakistani and 2 black African) completed the entire study. On average, they had lived in the city for 186.8 ± 50.7 months (15.5 years). All the analyses in this study were carried out on the 25 participants who completed the entire study. These 25 participants listed a total of 1291 network members. Where appropriate, we follow standard terminology in network science by referring to a participant as *ego* and network members as *alters*.

At month 4 of the study, participants took their final school exams (A-levels) and left the school. Of the 25 participants who completed all three waves of data collection, six of them stayed in the city and worked, not going to university (*non-university stayers*), eight went to one of the two universities in the city (*university stayers*) (all 14 continued to live at home with their parents), and 11 went to universities elsewhere in England (*leavers*).

The study was approved by University of Liverpool Ethics Board.

### Social Network Questionnaire

Participants were first asked to provide demographic information: age, gender, ethnic origin, and length of time living in the city. They were then asked to list all their known and living relatives, including both genetic and affinal kin. A list of relatives, with descriptions out to first cousin (e.g., your great-aunt is an aunt of your father or mother), was provided to help prompt recall of more distant relatives. Both genetic and affinal kin are referred to as *kin*. Participants were also asked to list all friends “for whom you have contact details and with whom you consider that you have some kind of personal relationship (friend; acquaintance; someone you might interact with on a regular basis at school, work, or university).” This section of the questionnaire had the heading “Friends,” and this term is also used here. The use of this term does not imply anything about the strength of the relationship between the participant and the friend, which is instead measured directly through a number of relationship quality indices.

To help prompt their memory, the participants were asked to look through any lists of addresses or phone numbers that they had (e.g., address books, email addresses, contacts list in mobile phone). For kin, participants were asked to provide the following details of the relationship: (1) type of relation (e.g., father, mother, sibling, cousin); (2) genetic relationship (genetic, step, adoptive, related by marriage); (3) nature of genetic relationship (maternal, paternal, or neither). For friends, participants were asked how long they had known the friend (in months). For both kin and friends, participants were asked how emotionally close they felt to the each network member on a scale of 1 (*someone you never see or hear from*) to 10 (*someone with whom you have a deeply emotional relationship; perhaps someone you might go to for advice or comfort in times of major emotional trauma or crisis*). For genetic kin, the coefficient of genetic relatedness (*r*) was calculated for each relative (0.5 for parents and siblings, 0.25 for grandparents, etc.). Affinal kin (in-laws) are by definition not genetically related to the participant, so their coefficient of genetic relatedness is zero.

Participants were then asked two questions designed to distinguish those strong relationships in the inner core of the personal network from the weaker relationships with other family and friends. We asked the participants to identify “all individuals from whom you would seek advice, support, or help from in times of severe emotional or financial distress.” We also asked participants to list, *in addition* to the network members listed in response to the first question, all individuals “whose death you would find personally devastating.” These two questions have previously been used to establish the “support group” and “sympathy group” layers of the social network, respectively (Binder et al. 2012; Buys and Larson 1979; Dunbar and Spoors 1995; Sutcliffe et al. 2012). In this study, the support group and sympathy group were combined and referred to as the *inner* layer (equivalent to Granovetter’s *strong ties*). All other alters were defined as the *outer* layer of the network (equivalent to Granovetter’s *weak ties*).

In examining how ties change over time, an important question that arises is how to characterize the strength of a particular tie. A factor analysis of different measures of interpersonal closeness identified two key components: “behaving close” and “feeling close” (Aron et al. 1992). In this study, we include measures for both of these components. In terms of “feeling close,” Marsden and Campbell (1984) examined a range of measures and concluded that a measure of the emotional intensity of a relationship is the best indicator of tie strength. Thus, we took emotional closeness, measured on a 1–10 scale, as an indication of the emotional intensity of the relationship. This measure is simple for the participants to use when rating a large number of network members, and the measure (or a similar one) has been used in a large number of previous studies by different research groups (e.g., Cummings et al. 2006; Hill and Dunbar 2003; Jeon and Buss 2007; Korchmaros and Kenny 2001; Roberts et al. 2009). We also used another well-established measure, the Subjective Closeness Index (Berscheid et al. 1989), but since this correlated strongly with the emotional closeness measure (*r* = 0.90, *p* < 0.001), we report only the latter.

To examine the “behaving close” component, we used two measures: frequency of contact and number of different activities done together. These were used as indicators of participants engaging in relationship maintenance behaviors. As in previous studies (Hill and Dunbar 2003; Roberts et al. 2009), participants listed how many days ago they last made contact with each network member, either face-to-face or by other means (e.g., phone, email). Communication frequency is associated with the strength of a relationship (Mok et al. 2007) and the probability of receiving support (Kana’Iaupuni et al. 2005). Participants were also asked how many different types of activities they had done together in the preceding 12 months at T1, and the past 6 months at T2 and T3 (the questionnaires at T2 and T3 were given six months apart). The activities listed were sport or physical activity (e.g., football, keep fit, mountain biking), leisure activity (e.g., shopping, going to cinema, going to see a gig), social activity (e.g., going to the pub, going round to their flat/house, meeting at a social event), work activity (e.g., going to classes or lectures, studying together, working together), and going on holiday (going away for more than one night). Participants indicated with a “yes” or “no” whether or not they had participated in each of these activities. The answers were then summed to give an *activity score*, which ranged between 0 (no activities) and 5 (all activities). Relationships in which more activities are done together tend to be more emotionally intense (Wellman and Wortley 1990) and more resistant to decay (Degenne and Lebeaux 2005).

The Social Network Questionnaire was completed by the participants at month 1 (T1), month 9 (T2), and month 18 (T3). At T2 and T3, the initials, gender, and nature of the family relationship of all the network members listed at T1 were provided to aid recall and ensure that participants provided updated information on emotional closeness and location about all the network members listed at T1. At T2 and T3, participants were also requested to provide information on any new friends they had made since last completing the questionnaire.

### Statistical Analysis

To examine how emotional closeness between participants and network members changed over time, we used hierarchical linear growth modeling, also known as multilevel modeling. In this study, 1291 network members were clustered within 25 participants, and the three time points were clustered within network members. Thus these data points could not be treated as independent samples in an ordinary least squares regression analysis (Bryk and Raudenbush 1992). Multilevel analysis is a modified form of multiple linear regression designed to deal with data with a hierarchical clustering structure and has been extensively used in analysis of personal network data (e.g., Gierveld and Perlman 2006; van Duijn et al. 1999; Wellman and Frank 2001).

For models 1–7, emotional closeness was the dependent variable and we used a three-level model structure. Starting at the lowest level of measurement, Level 1 represents time—the repeated observations of individual network members at T1, T2, and T3; Level 2 represents network member characteristics (e.g., alter’s gender, location) and tie characteristics (e.g., length of time known participant has known that network member); and Level 3 represents participant characteristics (e.g., ego’s gender, location, size of network). In model 8, change in emotional closeness was the dependent variable and so a two-level model was used: Level 1 was network member/tie characteristics and Level 2 was participant characteristics. For all models, we used a linear model structure since the dependent variable was continuous (emotional closeness or change in emotional closeness). Using a linear model structure allowed for comparability across the different models in this paper, and with previous literature (e.g., Cummings et al. 2006).

In models 1–7, the coefficients for the level 2 and level 3 variables represent cross-sectional associations. Thus, for example, a coefficient for participant gender and emotional closeness indicates whether, overall, there was a difference between the male and female participants in their emotional closeness to network members. The interactions with time represent variables that predict *changes* in emotional closeness over time. Thus, an interaction between time and participant gender indicates whether there was an effect of participant gender on how their emotional closeness with network members changed over the course of the study. All variables included in the construction of the models are listed in the Results section, below.

We followed the guidelines detailed by van Duijn et al. (1999) in guiding our selection of the models. Thus, we started with an empty model, including only the intercept and the error term for all levels. This gives an indication of the amount of variance present at the three levels. We then used a forward selection procedure (Bryk and Raudenbush 1992) involving three steps: (1) adding fixed explanatory variables (including interaction terms between them); (2) adding cross-level interaction terms; and (3) adding random intercepts, random slopes, and covariances between the random slopes. A summary of all models is provided in Table 1.

**Table 1:** Summary of all multilevel regression analyses, models 1–8

Model	Dependent variable	*N* of levels	Details of levels	Random effects
1	Emotional closeness	3	1: Participant characteristics 2: Network member 3: Time	Random intercept: Participant, network member Random slopes: Time Covariance between slopes and intercepts: First-order autoregressive structure (ARH1)
2	Emotional closeness to kin	3	1: Participant characteristics 2: Network member 3: Time	Random intercept: Participant, network member Random slopes: Time Covariance between slopes and intercepts: First-order autoregressive structure (ARH1)
3	Emotional closeness to friends	3	1: Participant characteristics 2: Network member 3: Time	Random intercept: Participant, network member Random slopes: Time Covariance between slopes and intercepts: First-order autoregressive structure (ARH1)
4	Emotional closeness to T1 friends at T1 and T2	3	1: Participant characteristics 2: Network member 3: Time	Random intercept: Participant, network member
5	Emotional closeness to T1 friends vs T2 and T3	3	1: Participant characteristics 2: Network member 3: Time	Random intercept: Participant, network member
6	Emotional closeness to kin at T1 and T2	3	1: Participant characteristics 2: Network member 3: Time	Random intercept: Participant, network member
7	Emotional closeness to kin at T2 and T3	3	1: Participant characteristics 2: Network member 3: Time	Random intercept: Participant, network member
8	Change in emotional closeness (T3 − T1)	2	1: Participant characteristics 2: Network member	Random intercept: Participant

In all models, maximum likelihood (ML) estimation was used, rather than restricted maximum likelihood (REML), as this allows comparison of the deviance of different models (Tabachnick and Fidell 2007). We used Schwarz’s Bayesian criterion (BIC) to compare the goodness-of-fit of the models, whilst correcting for the number of parameters used (Kuha 2004). The difference in deviance of two models can be used as a test statistic with a χ^2^ distribution, with the number of different parameters as the degrees of freedom (Hayes 2006). Based on the BIC criterion, we built the most parsimonious models possible, rather than including variables even if they did not significantly improve the goodness-of-fit of the models. To model the covariance, we used a heterogeneous first-order autoregressive structure (ARH1), which is particularly appropriate for longitudinal data because it assumes that the correlations between repeated measurements are highest at adjacent time points (Field 2013).

All continuous variables were Z-transformed to allow for comparability across variables measured on different scales. Kin and friendship network sizes were grand-mean centered (Models 2 and 3). This allows the intercept to be interpreted as the average outcome for each group, rather than using a score of zero, which is not meaningful for network size. Changes in activity score and changes in contact frequency were group-mean centered—a mean score was calculated for each participant, and the individual network members’ scores were centered around this participant mean (Model 8). This controls for individual differences in these variables between the participants. An alpha level of 0.05 was used for all statistical tests. All significance tests were two-tailed, except for the variance parameters. Since these are by definition non-negative, when testing the null hypothesis that the variance of a random intercept or random slope is zero, the alternative hypothesis is by definition one-sided (Snijders and Bosker 1999).

## Results

### Basic Network Properties at Time 1

The descriptives for the main variables are given in Table 2. Mean network size at T1 was 51.68 (*SD* = 27.58, *Mdn* = 46) with a range of 19–132. We first tested for normality using the Shapiro-Wilk test, which is more accurate than the commonly used Kolmogorov-Smirnov test (D’Agostino and Belanger 1990). Neither kin (Shapiro-Wilk *W*_30_ = 0.86, *p* = 0.001) nor friendship network size (*W*_30_ = 0.90, *p* = 0.007) were normally distributed, so nonparametric tests were used. We then tested whether network size varied according to gender and ethnic group. There was no significant difference in the size of the kin network between white (*Mdn* = 19) and black/Asian participants (*Mdn* = 14) (Mann–Whitney *U* = 65.5, *p* = 0.66). Although white participants (*Mdn* = 35) tended to have a larger friendship network than the Asian or black participants (*Mdn* = 23), the difference was not formally statistically significant (*U* = 99.5, *p* = 0.06). The difference in the size of the kin network did not differ significantly between male (*Mdn* = 12) and female (*Mdn* = 19) participants (*U* = 72.0, *p* = 0.10). Nor was there a significant difference in the size of the friendship network between male (*Mdn* = 29) and female (*Mdn* = 26) participants (*U* = 108.50, *p* = 0.88).

**Table 2:** Variables used in multilevel regression models predicting emotional closeness from network member characteristics, participant characteristics and time in study

Variable	Description	Mean	*SD*	Median	*n*
*Level 1: Network member /Tie characteristics*					
Gender	0 = Female; 1 = Male	0.42	(0.49)	0	1287
Location	0 = Same city as school 1 = Different city from school	0.67	(0.47)	1	1265
Relationship	0 = Family; 1 = Friend	0.61	(0.49)	1	1288
Relatedness (*r*)	Genetic relatedness	0.20	(0.16)	0.125	499
Network layer	0 = Inner; 1 = Outer	0.75	(0.43)	1	1
Time known (friends)	Length of time known (months)	58.88	(50.53)	36	789
Contact frequency (T1) ^a^	Number of days to last contact	28.41	(84.34)	1	789
Contact frequency (T2) ^a^	Number of days to last contact	69.31	(109.56)	22.50	694
Contact frequency (T3) ^a^	Number of days to last contact	128.78	(153.51)	27	725
Activity score (T1) ^a^	Number of different activities	2.00	(1.13)	2	789
Activity score (T2) ^a^	Number of different activities	1.25	(1.25)	1	739
Activity score (T3) ^a^	Number of different activities	0.97	(1.09)	1	716
*Level 2: Participant/Network characteristics*					
Gender	0 = Female; 1 = Male	0.52	(0.51)	1	25
Ethnic group	0 = White; 1 = Black or Asian	0.40	(0.50)	0	25
Location	0 = Same city as school 1 = Different city from school	0.96	(0.20)	1	25
Kin network size	Size of related network	20.04	(16.17)	13	25
Friend network size	Size of unrelated network	31.64	(18.07)	28	25
Time in school city	Months lived in school city	186.83	(50.65)	204	25
Participant location at T2	0 = Same city as school 1 = Different city from school	0.56	(0.51)	1	25
University at T2	0 = Not Univ.; 1 = Univ.	0.76	(0.44)	1	25

*Notes*: All measures are for Time 1 unless otherwise stated; *n* varies across variables owing to missing data

^a^ Contact frequency and activity score are for friends only, because these were the variables used in Model 8

- *H1: Do friendships decline in emotional intensity over time more than family relationships?*

**Fig. 1:**
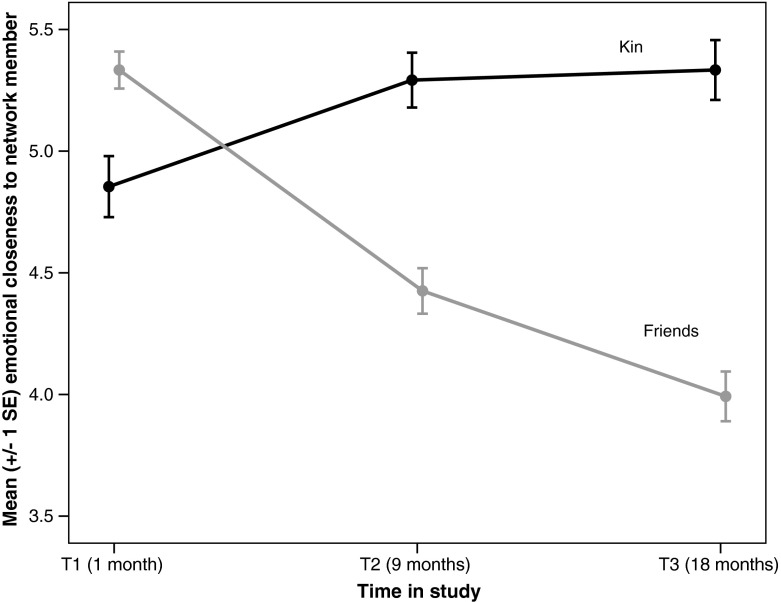
Mean emotional closeness of participants to network members by type of relationship. Note that “family” means extended family. Emotional closeness measured on a scale of 1–10, where 10 is “very close”

H1 predicted that friendships would decay more than relationships with family. Model 1 tested this by examining the effect of time on emotional closeness levels for family and friends. The type of relationship had a significant effect on how emotional closeness changed through time, as indicated by the significant interaction in the model between time and relationship type (see Table 3 and Fig. 1). The significant random effect of time indicates significant variation between the participants in the effect of time on changes in emotional closeness. Separate models were then used to examine how emotional closeness changed over time for kin and friends. There was a significant *increase* in emotional closeness for family (*b* = 0.27, *t*_542.10_ = 7.28, *p* < 0.001) and a significant *decrease* in emotional closeness for friends (*b* = −0.62, *t*_786.57_ = −15.23, *p* < 0.001). Thus, H1 was supported: friendships decayed more than relationships with family between T1 and T3.

**Table 3:** Model 1: Multilevel regression model predicting emotional closeness from relationship type and time

Predictors	Parameter estimate	SE	95% CI	
*Fixed effects*				
Intercept	4.68***	0.13	4.44	4.94
Time	0.27***	0.05	0.18	0.36
Relationship type	1.20***	0.16	0.87	1.51
Time × relationship type (kin/friends)	−0.90***	0.06	−1.01	−0.78
*Covariance parameters*				
Random intercept	5.68***	0.38	4.97	6.48
Random slopes (time)	0.29 ***	0.06	0.20	0.42
Covariance between intercept and slope (ARH1)	−0.27***	0.07	−0.40	−0.13

Table shows parameter estimates, standard errors and 95% Confidence Intervals (CI)

*Note*: The covariance between the random intercept and random slopes is modeled using a first-order autoregressive structure (ARH1)

*** *p* < 0.001

An alternative measure of relationship quality is whether alters moved between network layers. Compared with kin, friends were significantly more likely to move from the inner to the outer layer over the course of the study: of 145 kin in the inner layer at T1, 102 (70.3%) were still in the inner layer at T3, whereas only 69 of the 142 friends in the inner layer at T1 (48.6%) remained in the inner layer at T3 (χ^2^ = 14.10, df = 1, *N* = 287, *p* < 0.001). Thus, not only was there a quantitative decline in emotional closeness for friends over the course of the study, but also the relationships changed in a qualitative way.

**Fig. 2:**
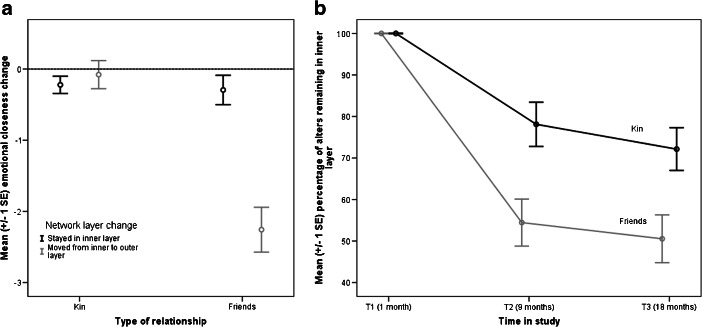
(**a**) Mean change in emotional closeness between T1 and T3 by type of relationship and position of network member in personal network at T3 (inner layer or outer layer). Chart shows network members who were in inner layer at T1 only. (**b**) Mean percentage of network members remaining in inner layer of personal network by time in study. Graph shows network members who were in inner layer at T1 only

We used separate multilevel models for kin and for friends to examine how these movements from the inner to the outer layer were associated with changes in emotional closeness. For simplicity, we report only whether there was a significant effect of time on emotional closeness from T1 to T3. For network members who stayed in the inner layer from T1 to T3, there was no significant change in emotional closeness (Fig. 2a). This was the case both for friends (*b* = −0.13, *t*_67.05_ = −1.32, *p* = 0.19) and for kin (*b* = −0.08, *t*_95.69_ = −1.47, *p* = 0.16). Moving to the outer network layer was associated with a significant decline in emotional closeness for friends (*b* = −1.22, *t*_76.93_ = −7.48, *p* < 0.001), but not for kin (*b* = −0.02, *t*_48.51_ = −0.15, *p* = 0.88). Note that the major movement of network members from the inner to the outer layer of the personal network occurred between T1 and T2, rather than between T2 and T3 (Fig. 2b). Of those network members who were in the outer layer at T1 (340 kin and 627 friends), just 40 (11.8%) kin and 42 (6.7%) friends moved into the inner layer at T3. Thus, it was significantly less common for network members to move from the outer to the inner layer of the network than from the inner to the outer layer (χ^2^ = 169.79, df = 1, *N* = 1254, *p* < 0.001).

- *H2: Does emotional closeness to family increase more in those who move further away?*

H2 predicted that whether a participant stayed in the hometown or moved away would have an effect on subsequent emotional closeness to friends, but not to kin relations. The results are presented in Table 4. In Model 2 (kin), time was positively related to emotional closeness: the emotional closeness of the participants to kin increased over the three time points in the study. In terms of network member characteristics, participants were emotionally closer to network members who were more closely genetically related to them and those who were in the inner network layer. Participants with larger kin networks tended to have lower emotional closeness to kin on average, whereas participants with smaller friendship networks tended to have lower emotional closeness to kin.

**Table 4:** Models 2 and 3: Multilevel regression models predicting emotional closeness from participant and network member characteristics and time

Model type	Model 2: Kin		95% CI		Model 3: Friends		95% CI	
*Fixed effects*								
Intercept	5.21 ***	(0.35)	4.53	5.91	7.32 ***	(0.33)	6.68	7.96
*Level 1*								
Time	0.55 ***	(0.12)	0.31	0.79	−0.52 ***	(0.09)	−0.69	−0.36
*Level 2 (network member /tie) variables*								
Genetic relationship (*r*)	0.93 ***	(0.17)	0.59	1.27	n/a			
Network layer at T1	−2.38 ***	(0.23)	−2.84	−1.94	−2.71 ***	(0.15)	−3.01	−2.41
Location	NS				−0.46 *	(0.19)	−0.59	−0.10
Gender	NS				−0.34 **	(0.13)	−0.59	−0.10
Time known (months)	n/a				0.27 ***	(0.06)	0.16	0.39
*Level 3 (participant/network) variables*								
Gender	1.17 ***	(0.18)	0.83	1.51	0.50 **	(0.18)	0.15	0.87
Ethnic group	1.42 ***	(0.18)	1.08	1.77	0.47 **	(0.14)	0.19	0.76
Mean genetic relationship	0.86 ***	(0.07)	0.72	1.00	n/a			
Mean time known	n/a				0.37 ***	(0.09)	0.20	0.54
Kin network size ^a^	−0.62 ***	(0.15)	−0.91	−0.33	NS			
Friendship network size ^a^	1.02 ***	(0.12)	0.79	1.26	NS			
Length of time lived in school city	NS				NS			
Destination after school (not Uni/Uni)	0.99 ***	(0.26)	0.49	1.50	0.78 ***	(0.22)	0.36	1.21
Participant location	1.01 ***	(0.23)	0.57	1.46	0.46 **	(0.15)	0.16	0.75
*Cross-level interactions*								
Time × relatedness	NS				n/a			
Time × network layer	0.26 **	(0.08)	0.11	0.42	NS			
Time × kin network size	0.29 ***	(0.04)	0.22	0.36	NS			
Time × time known (months)	n/a				NS			
Time × friendship network size	−0.31 ***	(0.04)	−0.39	−0.23	NS			
Time × participant gender	NS				0.36 ***	(0.08)	0.20	0.53
Time × Uni (not Uni/Uni)	−0.51 ***	(0.09)	−0.69	−0.32	−0.30 **	(0.09)	−0.48	−0.11
Time × destination	−0.71 ***	(0.08)	−0.87	−0.55	NS			
*Covariance parameters*								
Random intercept	2.42***	(0.32)	1.86	3.14	1.68***	(0.40)	1.06	2.67
Random slopes (time)	0.08	(0.05)	0.03	0.26	0.32 **	(0.09)	0.17	0.54
Covariance between intercept and slope (ARH1)	−0.38 **	(0.13)	−0.61	−0.10	−0.11	(0.21)	−0.49	0.29

Table shows parameter estimates (and SE) and 95% Confidence Intervals (CI)

*Notes.* NS refers to a variable that was not significant and thus not included in the final model. n/a refers to a variable that was not applicable to that model. The covariance between the random intercept and random slopes is modeled using a first-order autoregressive structure (ARH1)

^a^Grand-mean centered

* *p* < 0.05, ** *p* < 0.01, *** *p* < 0.001

**Fig. 3:**
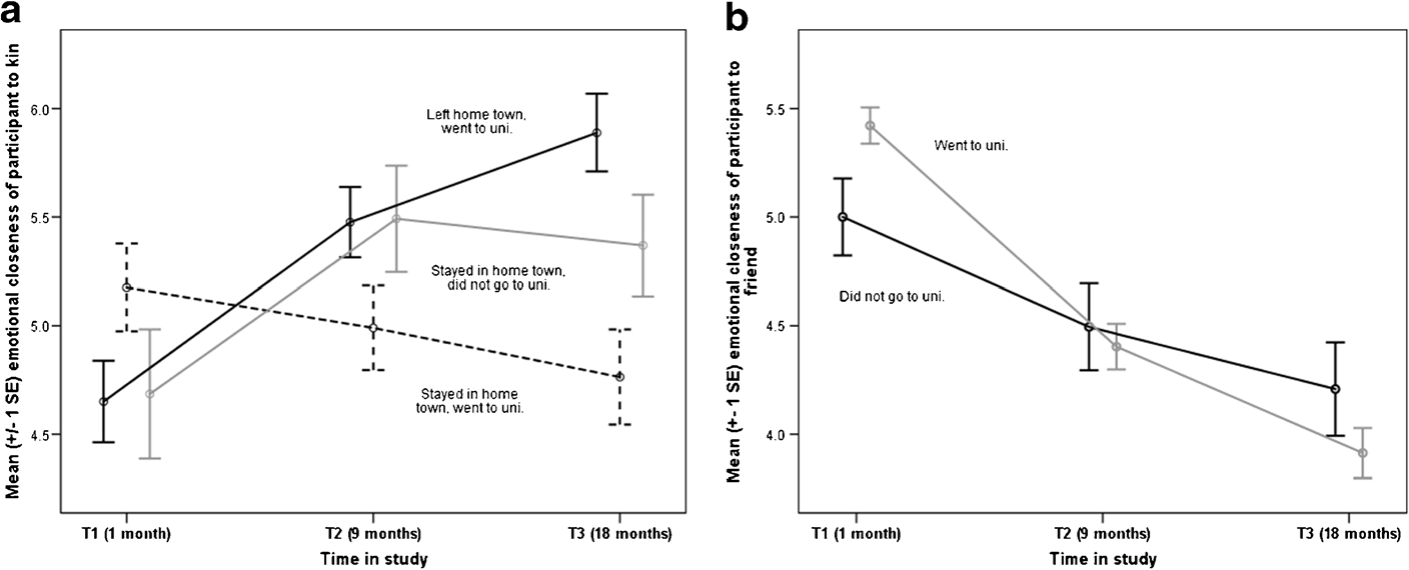
Mean emotional closeness to (**a**) kin and (**b**) friends by participants’ destination after school and university choice. Emotional closeness measured on a scale of 1–10, where 10 is “very close”

There was no significant interaction between time and level of genetic relatedness, so there was no tendency for the participants to become closer (or less close) over the course of the study to kin who were more closely related. If anything, kin at the outer layers of the personal network showed a greater increase in emotional closeness than those in the inner layers. However, time interacted with both the location of the participant and whether or not they went to university. Leavers, and non-university stayers, showed an increase in emotional closeness over time, whereas university stayers showed a decrease (Fig. 3a). Thus, H2 was not supported: the location of the student did have an effect on the emotional closeness to kin, albeit a rather complex one. In contrast with the cross-sectional associations reported above, the interactions between time and network size showed that participants with a larger kin network size had a greater increase in emotional closeness over time and participants with fewer friends had a greater increase in emotional closeness to kin over time.

- *H3: Are strong friendships more resistant to declines in emotional closeness?*

Model 3 (Table 4) explored the effect of moves and location in friends (H2) and tested whether stronger relationships were less likely to decay as a consequence of these kinds of stress (H3). The emotional closeness of participants to friends named at T1 decreased over the three time points in the study. Participants were emotionally closer to friends who were in the inner layer of the personal network, whom they had known for a long time, and who did *not* live in the hometown. This could be because those friends who did live in the city were simply casual friends that participants knew only through school. The participants were emotionally closer to female friends than male friends.

In contrast to Model 2, friendship network sizes were not significantly related to emotional closeness. There was also no significant effect of the amount of time the participant had been living in the hometown on the degree of emotional closeness to friends. There were only two significant interactions with time. First, participants who went to university showed a greater drop in emotional closeness to friends than those that did not go to university. Nonetheless, those that did not go to university still showed a significant drop in emotional closeness over time (Fig. 3b). Further, there was no significant interaction between leaving or staying in the hometown and change in emotional closeness. Thus, H2 was not supported: for those going to university, the location of the university did not impact changes in emotional closeness to friends. Second, female participants showed a greater drop in emotional closeness to friends over the course of the study than male participants.

H3 predicted that stronger relationships will be less likely to decay. However, there was no significant interaction between time and two important measures of the strength of the relationship at T1 (whether or not the friend was in the inner layer, and how long the participant had known the friend). Thus friendships that had lasted a long time, and friendships that participants defined as being in the inner layer of their personal networks, were *not* more resistant to decay in emotional closeness than more recent friendships or friendships in the outer layer of the network. The significant random effect of time indicates significant variation between participants in the effect of time on changes in emotional closeness to friends.

- *H4: Is the drop in emotional closeness to existing friends at Time 2 greater if more new friends have been added?*

**Fig. 4:**
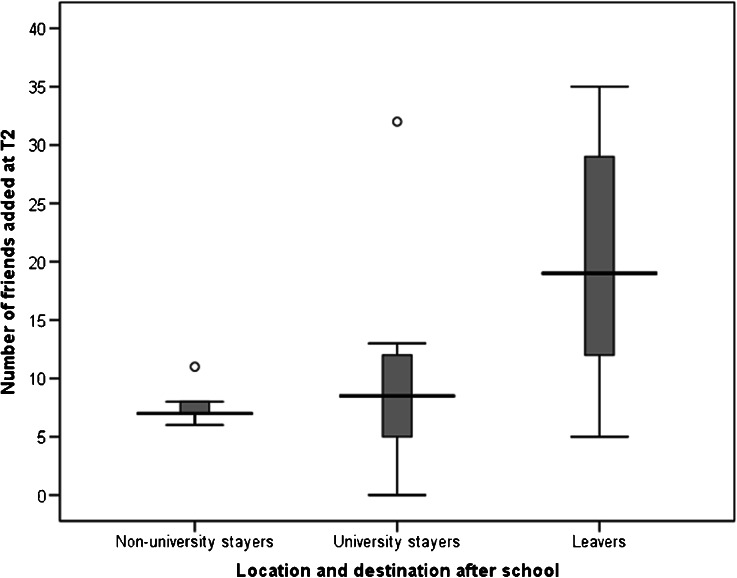
Number of friends added at T2 by whether participant went away to university (leavers), stayed in City A and went to university (university stayers), or stayed in City A and did not go to university (non-university stayers). Box plot shows median and interquartile range. Whiskers show minimum and maximum values, excluding outliers, which are shown as circles

The data on the number of friends added at T2 was not normally distributed (Shapiro-Wilk *W*_29_ = 0.85, *p* = 0.001), so nonparametric tests were used. Overall there was a significant difference among leavers, university stayers, and non-university stayers on the number of friends added (Fig. 4: Kruskal-Wallis test, *H*_2_ = 9.74, *p* = 0.008). Planned post hoc tests (with Bonferroni correction) showed a significant difference between leavers and university stayers (Mann Whitney *U* = 25.5, *p* = 0.01), but not between university stayers and non-university stayers (*U* = 28.5, *p* = 0.87).

We also examined the emotional intensity of these new friendships with those of existing friendships, and by destination. The data on emotional closeness of friends at T2 was normally distributed both for existing friends named at T1 (Shapiro-Wilk *W*_22_ = 0.98, *p* = 0.86) and new friends named at T2 (Shapiro-Wilk *W*_22_ = 0.94, *p* = 0.20), so parametric tests were used. There was no significant difference between the mean emotional closeness to friends named at T1 (*M* = 4.82, *SD* = 1.05) and that to the new friends named at T2 (*M* = 4.88, *SD* = 1.71; paired-samples *t*-test, *t*_21_ = −0.187, *p* = 0.85). Further, there was no significant difference in the emotional closeness to the new friends at T2 among non-university stayers (*M* = 4.79, *SD* = 1.59), university stayers (*M* = 3.95, *SD* = 1.56), and leavers (*M* = 5.66, *SD* = 1.68; one-way ANOVA, *F*_2, 19_ = 2.209, *p* = 0.14). Finally, there was no significant correlation between the number of friends added at T2 and the emotional closeness of these new friends (*r* = −0.065, *p* = 0.77). Thus the new friends added at T2 were no less emotionally close than existing friends, and participants who added more friends at T2 did not merely add lots of casual acquaintances, but friends of the same level of emotional intensity as existing friends.

**Figure 5:**
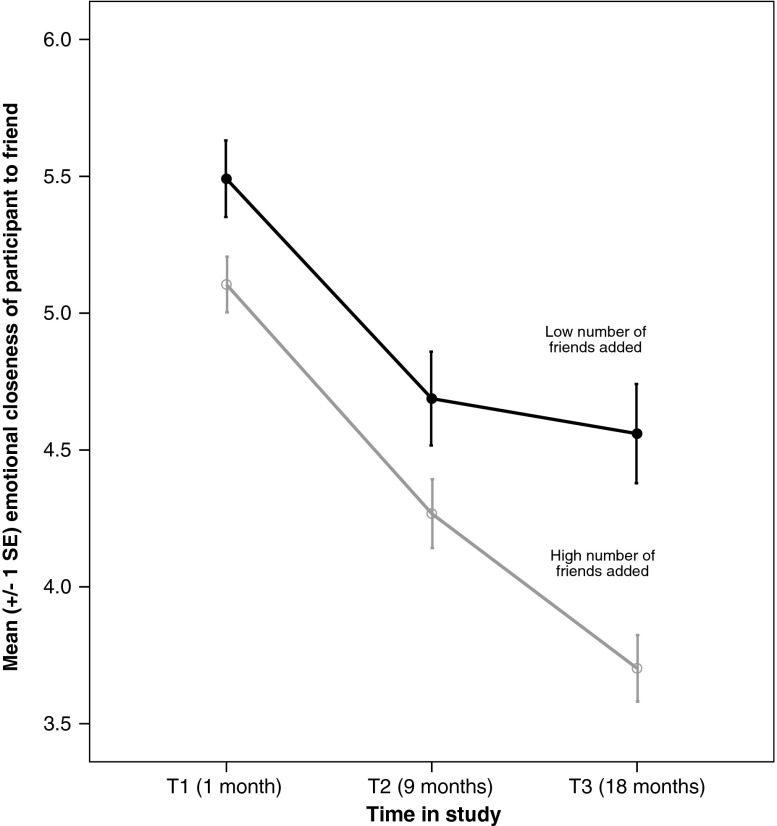
Change in emotional closeness to friends named at T1 based on number of new friends added at to network at T2. Emotional closeness measured on a scale of 1–10, where 10 is very close. Number of friends added median split into low (0–10) and high (12–35) for plotting only; model based on the continuous variable of number of friends added

Given that the emotional intensity of the new friendships is not related to the number of friends added, we focused subsequent models on the number of friends added at T2. H4 predicted that participants who added more friends to the network at T2 would show a greater decline in closeness to existing friends from T1. To test this, we examined how the number of friends added at T2 affected emotional closeness to existing friends named at T1. We used separate models to look at emotional closeness levels at T1 and T2 and then T2 and T3. Since the friends were added between T1 and T2, we expected the greatest impact on existing friends to be between T2 and T3. Between T1 and T2 there was no significant impact of the number of new friends added at T2 on the emotional closeness of the existing friends (Table 5, Model 4). However, between T2 and T3 (Model 5), there was a significant interaction between number of friends added and emotional closeness (Table 5, Fig. 5). Thus participants who added more new friends at T2 showed a greater decrease in emotional closeness to old friends than participants who added fewer friends at T2. The analysis was repeated for kin: between T1 and T2, and also between T2 and T3, there was a significant negative interaction between friends added at T2 and closeness to kin (Table 6, Models 6 and 7). Thus, H4 was supported: participants who added more friends to the network at T2 showed a greater decline in closeness both to existing friends and to kin.

**Table 5:** Models 4 and 5: Multilevel regression model predicting emotional closeness to T1 friends from number of friends added at T2 and T3

Predictors	Model 4 (T1 to T2)		95% CI		Model 5 (T2 to T3)		95% CI	
*Fixed effects*								
Intercept	6.15 ***	(0.14)	5.87	6.43	5.08 ***	(0.21)	4.67	5.50
Time	−0.82 ***	(0.08)	−0.98	−0.67	−0.31 ***	(0.07)	−0.46	−0.16
Friends added at T2	−0.42 **	(0.13)	−0.68	−0.16	0.11 †	(0.20)	−0.28	0.51
Time × friends added	0.04 †	(0.07)	−0.11	0.18	−0.22 **	(0.07)	−0.36	−0.08
*Covariance parameters*								
Random intercept	3.89***	(0.26)	3.41	4.43	5.48***	(0.33)	4.86	6.17

Table shows parameter estimates (and SE) and 95% Confidence Intervals (CI)

*** *p* < 0.001, ** < *p* 0.01, † Not significant

**Table 6:** Models 6 and 7: Multilevel regression model predicting emotional closeness to kin from number of friends added at T2 and T3

Predictors	Model 6 (T1 to T2)		95% CI		Model 7 (T2 to T3)		95% CI	
*Fixed effects*								
Intercept	4.29 ***	(0.17)	3.96	4.62	5.58 ***	(0.19)	5.21	5.95
Time	0.60 ***	(0.08)	0.44	0.75	−0.05 †	(0.06)	−0.17	0.06
Friends added at T2	−0.51 **	(0.17)	−0.85	−0.18	0.80 ***	(0.19)	0.42	1.18
Time × friends added	0.23 **	(0.08)	0.07	3.89	−0.41 ***	(0.06)	−0.53	−0.29
*Covariance parameters*								
Random intercept	6.63***	(0.47)	5.77	7.62	6.32***	(0.43)	5.54	7.22

Table shows parameter estimates (and SE) and 95% Confidence Intervals (CI)

** *p* < 0.01, *** *p* < 0.001, † Not significant

- *H5: How is the decline in emotional quality of friendships prevented?*

**Fig. 6:**
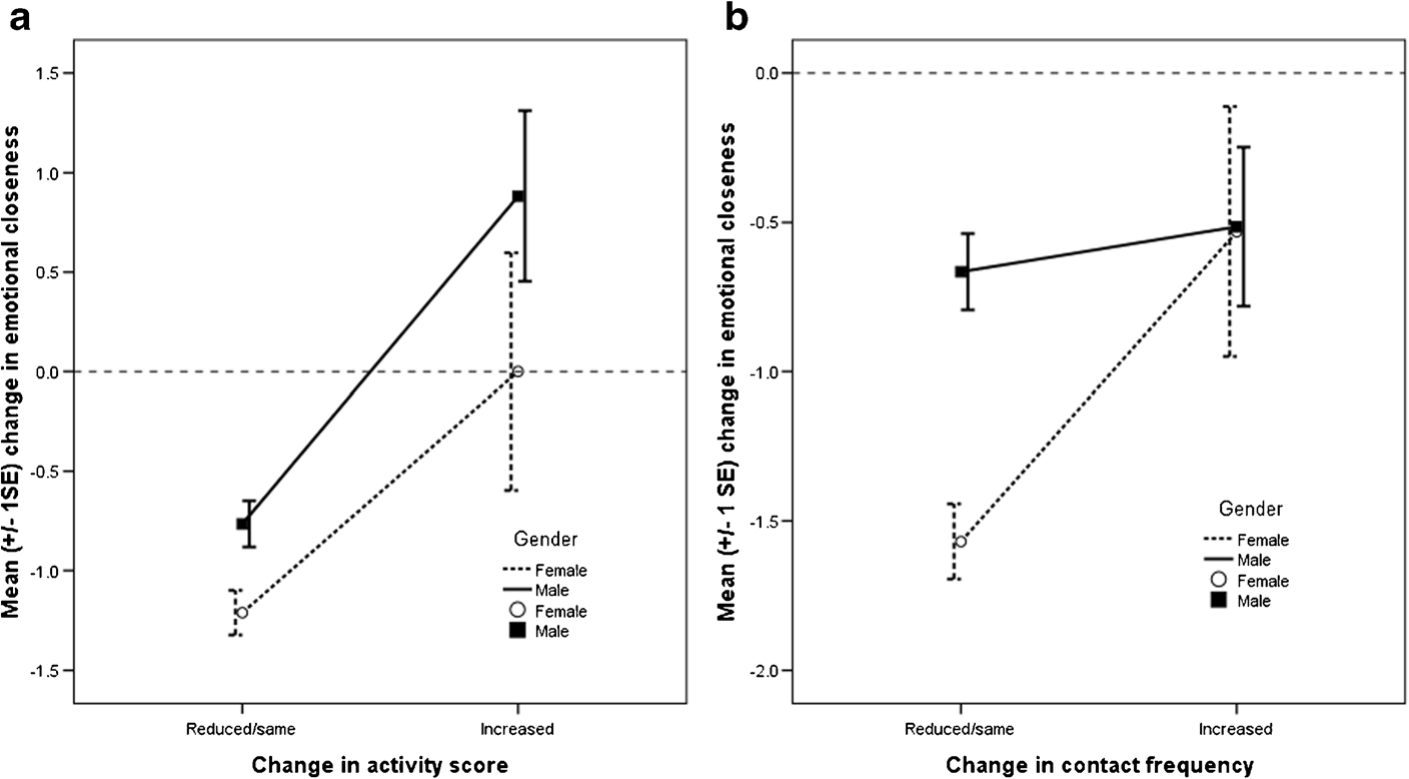
Gender differences in the impact on change in emotional closeness of a friendship of change in (**a**) number of different activities performed together and (**b**) change in contact frequency face-to-face or by phone. Activity score and contact frequency are split into dichotomous variables for plotting only; model based on continuous variable change in activity score

Finally, H5 predicted that interacting at a higher rate following physical or social separation would be required to prevent friendships declining in emotional quality. We restricted our analysis to friendships, because relationships with family showed no tendency to decline in quality with time or separation. Model 8 (Table 7) showed significant interactions between gender and change in activity score, and between gender and change in contact frequency. For males, increasing the number of activities done together was associated with an increase in emotional closeness between T1 and T3, whereas in females this effect was much less pronounced (Fig. 6a). In contrast, changes in contact frequency had a large effect on emotional closeness for females, but not for males (Fig. 6b). Including the gender of the network member, and whether or not the participant and network member were the same gender, did not affect the significance of these interactions, and these variables were not significant in themselves. H5 was thus vindicated, but with the caveat that the relationship maintenance processes involved showed a striking gender difference.

**Table 7:** Model 8: Multilevel regression model predicting change in emotional closeness (time 3 – time 1) to T1 friends from change in activity score and change in contact frequency

Predictors	Model 8		95% CI	
*Fixed effects*				
Intercept	−0.81 *	(0.35)	−1.55	−0.09
Ego gender	0.25	(0.51)	−0.80	1.30
Change in activity score^a^	0.38 **	(0.11)	0.16	0.59
Change in contact frequency^a^	−0.71 ***	(0.17)	−1.03	−0.38
Participant mean change in activity score	0.18	(0.24)	−0.31	0.68
Participant mean change in contact frequency	−0.20	(0.27)	−0.76	0.35
Gender × change in activity score	0.35 *	(0.15)	0.06	0.64
Gender × change in contact frequency	0.51 *	(0.25)	0.03	1.00
*Covariance parameters*				
Random intercept (participants)	1.12 **	(0.38)	0.58	2.20

Table shows parameter estimates (and SE) and 95% Confidence Intervals (CI)

^a^ Group-mean centered

* *p* < 0.05, ** *p* < 0.01, *** *p* < 0.001

## Discussion

In this study, we tracked the entire active personal network of 25 students over a period of 18 months as they made the transition from school to university or work. Our aim was to examine how the emotional closeness of the relationships between participants and the network members changed over the course of the study. Specifically, we tested five hypotheses regarding the nature of these changes.

The first hypothesis (H1) was unequivocally supported: there was a significant increase in emotional closeness to kin over the course of the study, as compared with a significant decrease in emotional closeness to friends. This result is in line with evolutionary theory, which predicts clear differences in behavior toward family members and friends (Hamilton 1964; Roberts 2010) and is consistent with previous studies that have found an increase in closeness to parents occurring during the transition to university (Kenny 1987; Pipp et al. 1985; Sullivan and Sullivan 1980). In contrast to previous studies, however, this study demonstrates that the beneficial effect of going away to university applies not just to close family members, but seemingly to the entire extended-family network. In terms of friends, the decrease in emotional closeness did not occur just with the relatively strong ties in the inner layer of the personal network, but also in the outer layers of the personal network (which have likewise not been included in previous studies). Thus even “weak” ties become weaker over time. This illustrates the fact that weak ties are not static; they too require a certain level of maintenance if they are not to decay (Burt 2002; Roberts 2010).

In terms of the effect of distance on changes in emotional closeness, leavers and non-university stayers showed an increase in emotional closeness to kin, whereas university stayers showed a decrease. One possible reason for this difference could be that university stayers find living at home more irksome than university peers who are living with other students, and this resentment produces a decrease in closeness to family. For friends, participants who went to university showed greater decreases in emotional closeness than those who did not go to university. However, there was no difference in changes in emotional closeness between students who went to university in the hometown and those who did not. This suggests that it is the mere fact of going to university, rather than going *away* to university, that impacts on old friendships. Thus H2 was not supported: there was an effect of distance on changes in emotional closeness to kin, but no effect of distance per se for friends.

H3 predicted that stronger relationships will be less prone to decay. We used three criteria for relationship strength: being in the inner layer of the personal network, genetic relatedness for kin, and time known for friends. H3 was not supported. There was no interaction between time and genetic relatedness; thus the increase in emotional closeness over the course of the study was not restricted to close family members. Further, kin in the outer layers of the personal network actually showed a *greater* increase in emotional closeness over time than those in the inner layer. In terms of friendships, even friends whom participants had known for a long time, and friends who were classified in the inner layers of the network, were not immune from the decline in emotional closeness during the transition to university. For friends (but not for kin), these declines in closeness were associated with moving from the inner layer of close friends to the outer layer of weaker ties. Thus, contrary to early theorizing (e.g., Wright 1984), close friendships are not self-sustaining but are in fact just as prone to decay with physical separation and the competition generated by opportunities to meet new people. H4 predicted that participants who added more new friends at T2 would show a sharper decrease in closeness to existing network members. This hypothesis was supported: between T2 and T3, participants who added more friends showed decreased closeness both to old friends and to kin. Finally, H5 predicted that friendships that were invested in more heavily would be more resistant to decay with time and distance. This too was supported, but with the caveat that the mode of interaction that worked best differed between the genders: for males, engaging in activities together seemed to be crucial, whereas talking to each other was critical for females.

This study has three main implications. First, it reinforces the conclusion that kin relationships are more stable than friendships (Burt 2000) and are more resistant to changes in location or circumstance, and it extends this finding to a wider range of ties than is typically studied. Simply feeling psychologically close to old friends does not prevent these friendships from declining in closeness over time. As Fig. 6 indicates, preventing a decline in closeness requires active maintenance in terms of communication (Oswald and Clark 2003; Cummings et al. 2006) or doing activities together (Degenne and Lebeaux 2005). Second, it provides further support for the notion of constraints on network size, as suggested by the social brain hypothesis (Dunbar 1998; Roberts 2010; Roberts et al. 2009; Sutcliffe et al. 2012). As one example of an archetypal life transition, the transition to university is associated with a decrease in emotional closeness to friends, and this effect is especially pronounced when a participant added more friends to his/her network at T2. Importantly, these new friends were as emotionally close as existing friends (see also Saramäki et al. 2014), and there was no relationship between the number of friends added and the emotional intensity of these friendships. Given the close relationship between contact frequency and emotional closeness (Hill and Dunbar 2003; Roberts and Dunbar 2011), this suggests a substantial time investment in these new friendships. Because time is an inelastic resource (Nie 2001), those participants who invested time and energy into making new friends appeared to do so to the detriment of existing friendships. Third, the effect of distance on social relationships appears to be more complex than previous studies have suggested. For kin, absence really did “make the heart grow fonder”: those who left their hometown showed a greater increase in emotional closeness than those who stayed. For friends, leaving home per se had no effect on the decline in emotional closeness; rather the effect is driven by the opportunity to meet new people that a change in circumstances provides. Overall, although distance generally may have a negative effect on closeness of social relationships (Mok et al. 2007; van Duijn et al. 1999), the effect of distance does not seem to apply equally to kin relationships and friendships.

One of the strengths of this study was that we collected information on the participants’ relationships before they underwent their life transition. Most studies of this type are retrospective, recruiting students after they have arrived at university: this inevitably means that much of the change in social relationships associated with the transition to university has already happened (see Fig. 3b), and as a result previous relationships may be overlooked. Further, the attrition rate in our study was exceptionally low for a study of this nature and length. Of the 30 students who started the study, 25 (83%) completed all waves of data collection. This compares very favorably with the equivalent figures from comparable longitudinal studies: 79% (Hays and Oxley 1986), 67% (Paul and Brier 2001), 55% (Oswald and Clark 2003), and 23% (Cummings et al. 2006). High attrition rates on longitudinal studies can have serious consequences in terms of drawing valid conclusions from the data (Jeličić et al. 2009).

Our study does, nonetheless, have some limitations. First, the sample size is modest. However, multilevel modeling allowed full use to be made of the data, with the analysis being carried out at the level of the 1291 network members (alters) rather than just the 25 participants. Second, no interviews were carried out with the participants. Interviews can provide valuable help in interpreting the statistical findings in longitudinal studies (e.g., Buote et al. 2007; Lubbers et al. 2010). Third, the participants were all around 18 years of age, and thus the extent to which the patterns found in this study would also apply to a broader age range, and to other life transitions (such as moving to another area, being divorced, or changing jobs), needs further research. Nonetheless, the results reported here are broadly in line with previous research, which has shown a greater stability in family relationships as compared with friendships (Burt 2000; Milardo et al. 1983; Morgan et al.1997; Wellman et al. 1997). Thus, although this study focused on a particular age group undergoing a particular transition, we expect the key findings—that even close friendships require active maintenance, whereas family relationships are more resistant to decay—to be replicated in studies of other types of transitions. Finally, participants were asked to recall detailed information for a large number of network members, and this inevitably raises questions about the reliability of recall. To counteract this risk, the questionnaire was designed to encourage accurate recall by providing participants with the details of all network members named at T1 for the T2 and T3 questionnaires and only asking participants to respond on a yes/no basis in terms of the different activities done. Nonetheless, detailed diary studies have shown that these types of social network questionnaires are reliable at capturing relationship change over time, including changes in contact frequency and changes in network size (Asendorpf and Wilpers 1998).

In terms of future work, both the Relationship Investment Model (Rusbult 1983) and the notion of constraints on network size deriving from the social brain hypothesis (Dunbar 1998; Roberts et al. 2009) argue that one of the key factors explaining the decline in old friendships is the time and energy put into forming new friendships (see also Miritello et al. 2013; Saramäki et al. 2014), which inevitably takes away time and energy that could have been invested in maintaining old friendships. Some work has been done in this area (e.g., Milardo et al. 1983), but relatively little is known about time budgets as a form of social capital. How much time do people actually spend socializing, both face-to-face and not? How is this time divided up across the social network as a whole? How does individual variation in time spent socializing, and the type of socializing, affect the way relationships with kin and friends change over time? New forms of digital technology mean that people increasingly leave a digital trace of their communication and activity (Eagle et al. 2009; Lazer et al. 2009), and this may enable these questions to be addressed more effectively and in more detail than is possible when relying on time-consuming questionnaires.

We have not explored the extent to which individual differences in personality might be related both to the properties of social networks and network change over time. Extraverts have larger personal networks (Pollet et al. 2011) and are more effective at building up a new social support network in a new country (Furukawa et al. 1998). Extraverts also participate in more social activities and have a higher interaction rate with others than introverts (Argyle and Lu 1990; Asendorpf and Wilpers 1998), suggesting that they may be more successful at maintaining friendships over time. This would particularly be the case if extraverts chose to devote more of their free time to social activities, rather than more solitary pursuits. Extraverts may also be more likely to leave home for university and thus have the opportunity to make new ties. Future work could usefully explore the strategies extraverts use to manage the trade-off between building up new friendships and maintaining old friendships during periods of transition, and whether these strategies simply lead to larger personal networks or also to more emotionally intense ties with network members (Pollet et al. 2011).
